# Learning Pathways and Students Performance: A Dynamic Complex System

**DOI:** 10.3390/e25020291

**Published:** 2023-02-03

**Authors:** Pilar Ortiz-Vilchis, Aldo Ramirez-Arellano

**Affiliations:** 1 Sección de Estudios de Posgrado e Investigación, Escuela Superior de Medicina, Instituto Politécnico Nacional, Mexico City 11340, Mexico; 2 Sección de Estudios de Posgrado e Investigación, Unidad Profesional Interdisciplinaria de Ingeniería y Ciencias Sociales y Administrativas, Instituto Politécnico Nacional, Mexico City 08400, Mexico

**Keywords:** learning pathways, learning performance, complex network, deep learning

## Abstract

In this study, learning pathways are modelled by networks constructed from the log data of student–LMS interactions. These networks capture the sequence of reviewing the learning materials by the students enrolled in a given course. In previous research, the networks of successful students showed a fractal property; meanwhile, the networks of students who failed showed an exponential pattern. This research aims to provide empirical evidence that students’ learning pathways have the properties of emergence and non-additivity from a macro level; meanwhile, equifinality (same end of learning process but different learning pathways) is presented at a micro level. Furthermore, the learning pathways of 422 students enrolled in a blended course are classified according to learning performance. These individual learning pathways are modelled by networks from which the relevant learning activities (nodes) are extracted in a sequence by a fractal-based method. The fractal method reduces the number of nodes to be considered relevant. A deep learning network classifies these sequences of each student into passed or failed. The results show that the accuracy of the prediction of the learning performance was 94%, the area under the receiver operating characteristic curve was 97%, and the Matthews correlation was 88%, showing that deep learning networks can model equifinality in complex systems.

## 1. Introduction

Complex systems comprise interactions among many elements [1]. This kind of system could be physical, biological or social [2]. The emergence is a central feature of a complex system; the interaction of lower level elements of the system moulds the higher-order level patterns. Furthermore, examining the lower elements separately cannot give insight into how the system behaves at a macro level [2,3,4]. Hence, complex system-science focuses on these interactions instead of studying the elements themselves [2,5].

A complex network is a good representation of a complex system [6,7]. In educational research, Ramirez-Arellano [8] modelled student–Learning Management System (LMS) interactions with a network representing the individual learning pathways. These pathways were used to construct a collective network, which is a non-additive system. This means that the resulting collective network was not equal to the sum of individual ones. The individual learning pathways were mostly linear with some bifurcations; meanwhile, the collective learning pathways were fractal. Thus, focusing only on isolated components cannot explain the behaviour of the whole system [9].

Previous research has focused on studying learning pathways from the point of view of learning analytics and machine learning to personalise the sequence of learning elements or courses. Furthermore, network-based models are employed to extract network features (such as centrality measures) or to retain the relevant nodes and links by computing the maximum spanning trees or detecting communities. The objective of those studies was to classify learning performance using machine learning techniques. Learning analytics and network-based approaches have focused on exploring individual behaviour and learning pathways but have failed to conceptualise the phenomena as a complex system.

This research aims to provide empirical evidence that student learning pathways have the properties of emergence and non-additivity from a macro level; meanwhile, equifinality (same end, quantified by final grade of learning process but different learning pathways) is presented at a micro level. Moreover, the individual learning pathways and the relationship of their components were analysed to classify learning achievements. Furthermore, it was shown that the computation of online learning rate (OLR) on the individual pathways was lower correlated with the final grade, as computed on the collective learning pathways. The following research questions are stated:

R1. Does a strong relationship between OLR and learning performance emerge when analysis from a micro to a macro level is performed?
R2. Can the equifinality property in an individual learning pathway be modelled to classify the learning performance?
R3. Are the student’s learning pathways a complex system?

The remaining sections of this article report the related work and provide the learning pathways preliminaries and tools rooted in the complex network research. Then the methodology, results and discussion are described. Finally, the conclusions are presented.

## 2. Related Work

Learning pathways are dynamic trajectories or learning routes that could be different but end in the “same” place regarding learning achievement. Previous works have analysed the learning pathways of blended learning as a collective behaviour [8,10]. However, the analysis of the behaviours of individual agents is necessary [11,12]. These studies have shown that learning pathways have the emergence property. This means that analysing individual learning pathways—modelled as networks—cannot provide insight into their impact on learning achievements. On the contrary, collective pathway topology differs for students who pass compared to those who fail a course.

The complexity in learning pathways is due to individual experiences, such as a student’s motivation, emotions, engagement, cognition and metacognition that change over time [8,10,13]. These variations provoke the system’s elements to change their relationships to raise self-organisation and adaptive behaviour [6]. Linear models have been extensively employed in educational research and can model complexity together with non-linear approaches [4]. An example is a nonlinear fractional model to quantify the learning acquisition from the collective learning pathways [10]. The fractal dimension and ν parameters of this fractional model were estimated by multiple linear regression with behavioural and emotional engagement and disaffection as independent variables. These linear regressions explained 68% and 78% of the variance of the fractal dimension and ν, respectively.

From a learning analytics point of view, the learning pathways were grouped into self-directed and teacher-directed. The findings suggest that students who are free to review the learning content have a low level of engagement and need more scaffolding, while those who follow a predefined path show a higher level of engagement [14,15]. A similar study [16] found that participants in a massive online open course preferred self-directed pathways, and those with high self-regulated learning had a single entry pathway for courses. Moreover, in teaching biology, the student learning outcomes of students who followed a learning pathway were higher than those who learned from a traditional face-to-face course [17].

Personalised education has been attempted by providing individual learning sequences with information technology that analyses historical data and then suggests the next step in learning; such systems can be based on rules [18,19] or can integrate expert knowledge [20]. The results suggested that personalised learning pathways enhance learning outcomes [20]. A network-based learning path recommendation approach constructs course and learner networks; the latter is used to guide the pathway recommendation based on the similarity of the students’ learning performance. The course–course network is the basis for recommending appropriate courses in different scenarios, such as a student who has not been enrolled in any course [21].

Furthermore, learning pathways are modelled as a complex network, each node represents a course, and the Pearson correlation coefficient quantifies the relationship between courses. Based on the strength of the correlation, a network is constructed. Then the nodes with a correlation value above a given threshold are retained in the network. By varying the threshold, the resulting maximal spanning tree has features such as degree distribution, the clustering coefficient, and betweenness, and average paths centrality are analysed [22,23]. Following a community analysis on networks where each node was a student, the weight edges represented the similitude of the students’ learning behaviour; Mai et al. [24] discovered the communities; then the number of learning materials and the number of transitions were extracted. The communities with the highest and lowest average learning performances significantly differed in the number of learning materials reviewed. Moreover, the information extracted from communities was employed to build several machine learning models to classify students as passed or failed. The resulting area under the receiver operating characteristic curve of classification was 80% [24].

The previous works reported in this section had two aims: to recommend the next element of the learning path (courses or learning material) and to classify the learning performance based on the individual learning pathways. The recommendation employs rule and knowledge inferences models, or modelling the course and students’ similarity based on network. Moreover, network-based models were employed to extract network features (such as centrality measures and the number of learning materials reviewed) or retain the relevant nodes and links by computing the maximum spanning trees or detecting communities. The main objective of those approaches was to classify the learning performance using machine learning techniques. All these approaches focused on analysing individuals’ behaviour and learning pathways but failed to conceptualise the phenomena as a complex system. Furthermore, Ramirez-Arellano, Sigarreta Almira and Bory-Reyes [10] and Ramirez-Arellano [8] have shown the emergence of the fractality from collective learning pathways but have not studied the individual components to obtain evidence of non-additivity and equifinality, which are features of dynamic complex systems.

## 3. Preliminaries on Learning Pathway Networks

### 3.1. Construction of Learning Pathway Networks

**Figure 1:**
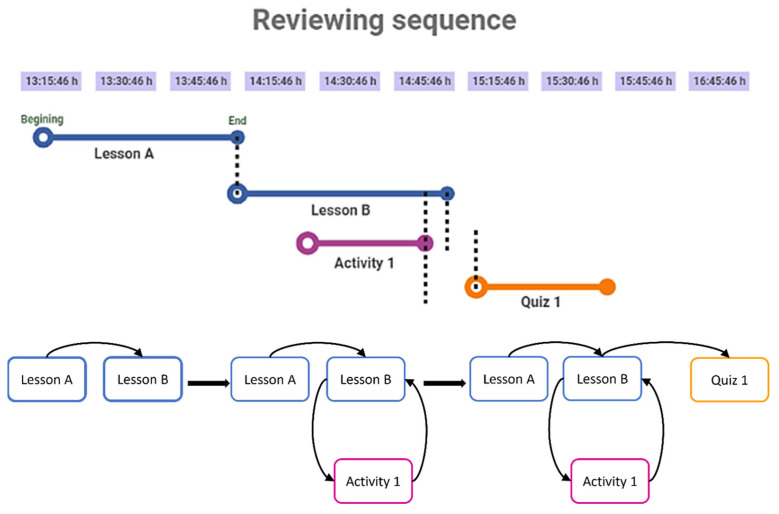
The individual learning pathway network construction from log files.

Individual and collective networks are constructed based on learning record history obtained from Moodle log files. This information includes lessons, quizzes, learning activities and examinations, chronologically ordered (from the beginning to the end); as shown in the top of Figure 1. Hence, this approach captures the dynamic of the system across time. For example, the first learning event (lesson A) is followed by the second (lesson B). This means that an arc from activity A to lesson B was added. Then, activity one was opened when lesson B was still being reviewed; hence, an arc from lesson B to activity one was drawn. Since activity one was finalised before lesson B ended, an arc from the first to the second was added. Quiz one was solved, and it was the final event connected to lesson B. For further details, the Supplementary Material contains an example of the implementation of the network construction algorithm [8].

**Figure 2:**
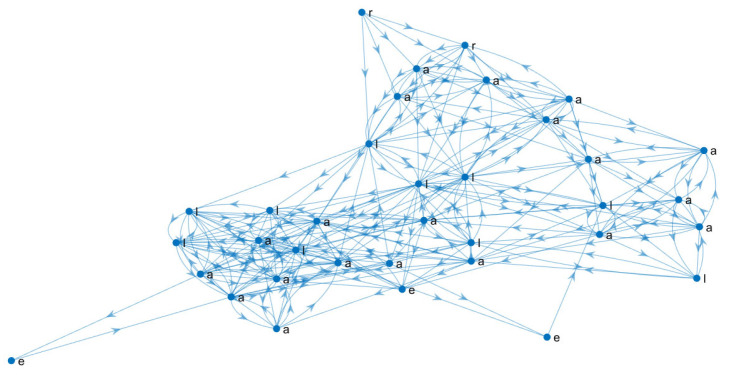
The collective learning pathway network from 42 students enrolled in Mathematics applied to Biological Science from January–July 2020 semester. a = activity, e = examination, l = lesson, r = reading.

The adjacency matrix of each personal learning network can be put together to build collective networks by graph union operation. For example, let *LP_1_* and *LP_2_* be the adjacency matrix of two individual learning pathways. To combine them, the “or” matrix operation denoted by *LP_1_*|*LP_2_* is defined as 1 if *LP_1_*(i,j) ≠ 0 or *LP_2_*(i,j) ≠ 0; it is defined as 0, otherwise. This operation can be repeated for every student enrolled in the course to obtain a collective learning pathway network. Since the individual learning pathways follow different combinations in the sequence of learning resources, the collective learning pathways are complex networks, as shown in Figure 2. The direction of the arcs indicates the order in which the resources were reviewed.

### 3.2. Extraction of the Relevant Nodes of Learning Pathway Networks

The topology of the collective pathway networks has been analysed in [8,10], and the findings show that these networks are fractal. This feature is the cornerstone of extracting the relevant nodes by identifying the network boxes [25]. Then, the nodes with the highest betweenness are removed to fragment the network. The resulting list of nodes contains those identified as relevant. The nodes that are disconnected by removing the nodes with the highest betweenness are not considered relevant. In a fractal network, the boxes contain a hub (the node where several nodes are connected), and those boxes usually are connected to other boxes by the hub (assortativity). Hence, the hub nodes are expected to have a high betweenness degree, and removing these vital nodes leaves the “satellite” nodes isolated from the rest of the network.

**Figure 3:**
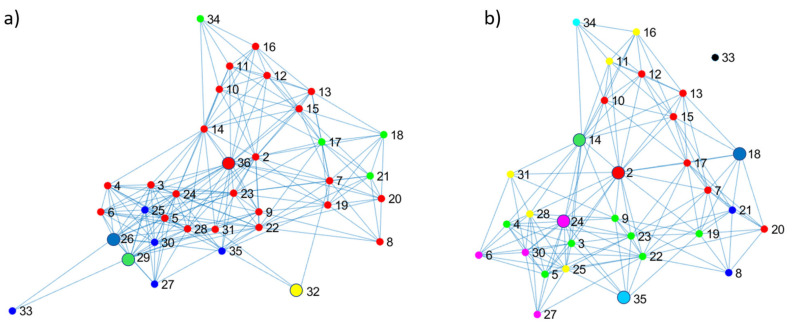
Extracting relevant nodes by computing the boxes to cover the learning pathway network of the January–July 2020 semester. (**a**) The minimum boxes to cover the network. (**b**) Nodes with the highest betweenness in each box are deleted, which are nodes 36, 29, 26 and 32.

An example is shown in Figure 3. The directed arcs are transformed into undirected ones before applying the procedure. Then the minimum boxes to cover the network are computed, as shown in Figure 3a. Note that nodes in the same colour belong to the same box. For each box, the node with the highest betweenness is deleted, which are nodes 36, 29, 26 and 32 in our example (and are big nodes, as shown in Figure 3b). The highest betweenness value computed in the whole network differs when computed on the boxes extracted as subnetworks. For example, the highest betweenness value of the network is of node 2 (51.93) but differs when the computation is carried out in the red box (42.63 for node 36 and 25.1 for node 2); hence, node 36 is removed. The node with the highest betweenness is removed from the remaining boxes (green, blue, yellow), deleting nodes 29, 26, and 32. After this, node 33 was disconnected, so it was not considered a relevant node. Next, the previous steps are repeated in the network, as shown in Figure 3b, adding 2, 14, 24, 18 and 35 to the list. The box covering and node removal steps are repeated on the resulting network(s) until every node has a neighbourhood. If two or more disconnected subnetworks are produced after node removal, the process is carried out on the subnetwork that has the higher number of nodes.

The fractal approach outperforms degree, betweenness, and PageRank methods. For further details on the fractal approach, see [25]. The relevant nodes of individuals’ pathways are ordered sequences (from highest to lowest relevance) that can be compared to those from the planned pathway. In other words, we compare how the students browse the material with how the faculty members expect it to be browsed.

**Figure 4:**
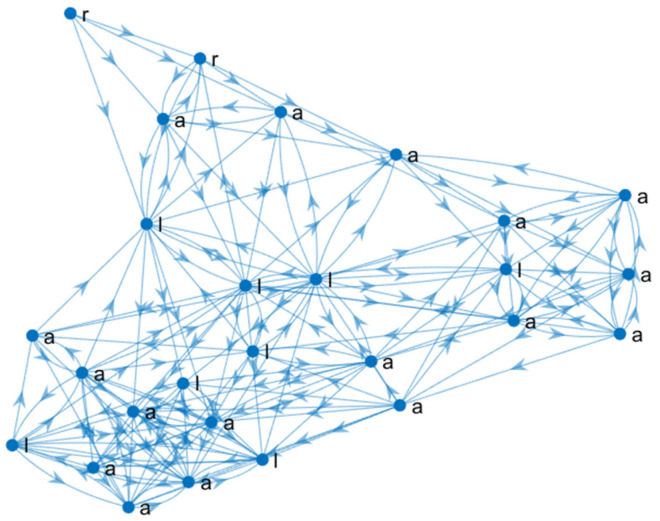
The relevant learning pathways are extracted by relevant node identification from the January–July 2020 semester. a = activity, e = examination, l = lesson, r = reading.

Extracting vital nodes can identify the relevant learning pathways in collective and individual learning networks. Once a list of nodes is obtained, the subnetwork formed by those nodes and their respective arcs can be extracted; an example of a relevant learning pathway is shown in Figure 4. The resulting network summarises relevant learning pathways and contains fewer nodes and arcs than the original one, as shown in Figure 2 and Figure 4. The relevant node identification produces an ordered list (from the most relevant to the least) that, in practice, is a subset of the original ones. This is an advantage over other node ordering methods, such as topological sort. Note that the examinations (e) of the network in Figure 2 were not relevant, so they were not included in the network in Figure 4.

### 3.3. Online Learning Rate

The online learning rate is defined as follows [8]:

(1) $$1-\frac{2\int_{l=1}^{\Delta }N_{b}(l)dl}{n^{2}}$$

where *n* is [M1] [US2] the number of nodes, Δ is the diameter plus 1 of the network, and *N_b_(l)* is the minimum number of boxes to cover the network. For a detailed description of the box-covering algorithm, see [26]. The OLR can be computed on individual and collective networks. The OLR is a normalised measure that can be compared among different learning pathway networks where one means the fastest learning rate and zero is the slowest.

The OLR computes the number of sessions (the uninterrupted time when students browse the learning material) necessary to cover all the learning material in the network. The rationale is varying the length (*l*) of the sessions. For example, given a network, as shown in Figure 3, the length of the session is represented by the diameter of the boxes (*l*) to cover the network using the box-covering algorithm. Thus, for a session length of *l = 1,* the number of sessions (*N_b_*) equals the number of nodes (*n =* 35; note that node enumeration starts from 2). In other words, reviewing the material at the speed of sessions of size one will take 35 sessions to complete the course. Now the speed is five (*l* = Δ = 5)–which means sessions of size five (box size)—hence, the entire network is covered by a session (one box) that includes all the learning material (nodes). It means that *N_b_* is computed with boxes of diameter *l =* Δ; thus, *N_b_* = 1. The box-covering implementation in the Supplementary Material obtains the *N_b_ for l = [2,* Δ−*1]*. When the student reviews the material in a linear pathway, the resulting plot of *l* vs. *N_b_* is similar to a straight line with a slope of −1/2.

## 4. Method

### 4.1. Participants and Context

The participants in this study were enrolled in a blended course, “Mathematics applied to Biological Science”, offered from 2019 to 2022. The number of university students was 424, with 201 males and 223 females. This course is included in a Mexican university bachelor’s degree program. It contains online lectures complemented with lessons, videos, tutorials, readings and learning activities delivered by Moodle. A new lecture is delivered weekly. The students’ materials (lectures and other resources) are available until the semester ends. Due to the COVID-19 pandemic, the weekly face-to-face sessions were supported via videoconference for 2020 and 2021. In the first semester of 2022, the session returned to the classroom. These sessions focused on solving individual concerns and giving deep explanations of learning activities and student feedback if necessary. The online lecture and its face-to-face session were scheduled for the same week.

The lecture articulates how the learning activities and other resources should be reviewed. It contains a linear pathway designed by the faculty members. Before the beginning of each semester, the faculty members revise and update each lesson. This update includes new materials, such as learning activities and readings. Moreover, the learning pathways can be updated by moving or deleting some resources. The lesson, examinations and learning materials are only available through Moodle, and students were not permitted to download them. Three examinations and learning activities were included in computing the students’ final grades. The final grade was the operationalisation of the student’s learning at the end of the course. Thus, achieving the same learning performance following different learning pathways shows equifinality. The learning activities were to be turned in by the following week; late deliveries were not permitted. The students received feedback for all uploaded learning activities. All students and teachers were informed of the research objectives and voluntarily participated. Both agreed that their anonymised information would be used for this research [27].

### 4.2. Analysis of Emergence

**Figure 5:**
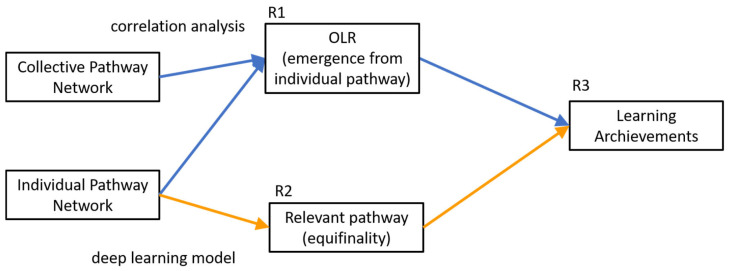
The overview of the method to answer the research questions.

The collective and individual learning pathway networks were constructed following the previous approach. The partitioned collective networks included all the students enrolled in the semesters, and they were constructed according to the final grade (passed or failed the course), as shown in Table 1. A student failed the course if he/she received a final grade below six. The Equation (1) computed the OLR in ten collective networks (partitioned) and 454 individual ones. The Supplementary Material contains an implementation and an example of the box-covering algorithm [26]. Using this approach, the improper integral $\int_{l=1}^{\Delta }N_{b}(l)dl$ can be approximated by the numerical integration of the point *l* vs. *N_b_*. The Mann–Whitney U test was performed on OLR (computed in collective and individual networks) to determine if the students who failed the course obtained a lower OLR score than those who passed. Moreover, the OLR and final grade correlations were analysed using the partitioned collective network. Moreover, this analysis was performed using all individual networks. These analyses of individual and collective networks seek to answer research question one, as shown in Figure 5.

### 4.3. Analysis of Equifinality

The relevant nodes extracted from the individual learning pathway network by fractal approach were compared with the expected learning pathways (the expected sequence to review the course content). Since both represent the lecture, learning activities and examinations, each sequence was encoded as a string, such as RLP = “l1,a3,e1” where l = lesson, a = learning activity and e = examination. Let ELP = “l1,a1,e1” be the expected learning pathway, then the Levenshtein distance [28] quantifies the total number of operations necessary to transform RLP into ELP. In the example, the student’s learning pathway differs from the expected by one since he/she solves activity three instead of one (the number 1 needs to be replaced by 3 in LP to become ELP). The Levenshtein distance was computed by semester since the ELP changed as faculty members revised and updated the course material. The Levenshtein distance was analysed by the Mann–Whitney U test to determine how far the students’ learning pathways were from the expected one and if there was a significant difference in their learning performance.

**Figure 6:**
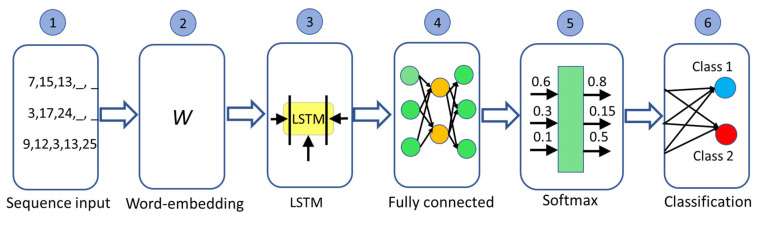
The architecture of the LSTM network.

Similarly, the encoded relevant nodes of individual learning pathways extracted by fractal approach and topological sort were employed to train and test a long short-term memory network (LSTMN). For this purpose, each relevant node sequence was treated as a sequence of “words”, where each word is the encoded node as in the previous example. Several students’ learning pathways differed in the sequence of nodes, but these students reached the same learning performance, showing equifinality. Based on this property, the LSTMN can classify the learning achievements to answer research question two, as shown in Figure 5. The architecture of the LSTMN is shown in Figure 6.

The “words” in each learning pathway sequence were encoded as integer numbers according to the vocabulary constructed from all individual learning pathways. The first layer received a one-dimensional sequence of these integer numbers. The word-embedding layer (dimension = 50) maps word indices to vectors that feed up to the LSTM layer. The LSTM layer contains 100 hidden units, and its output is the input of the fully connected layer. It connects all of the inputs to the outputs with weights and biases. The classification layer computes the cross-entropy loss for passed and failed, to choose the lowest value. The evaluation of the classification was performed using a ten-fold cross-validation technique. The LSTM network was implemented in MATLAB, and all the computations were performed on CPU Intel Core i7 9700, 64 Gb RAM, and GPU GeForce RTX 3090 with 24 Gb RAM.

## 5. Results

### 5.1. The Emergence of OLR and Learning Performance Correlation

**Figure 7:**
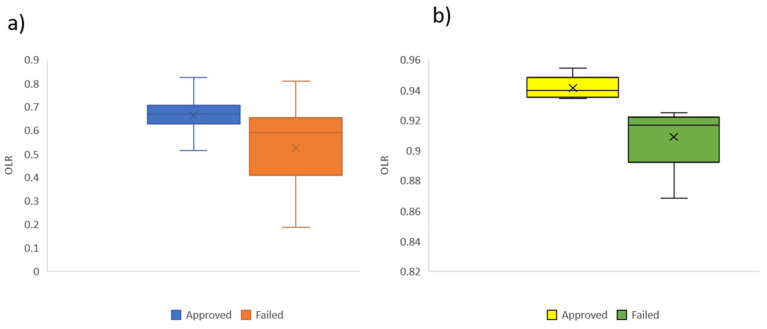
The significant difference in OLR by learning performance of individual learning pathways (**a**) and collective ones (**b**).

The Mann–Whitney U test on OLR was carried out in the ten partitioned learning pathway networks in Table 1. A significant difference was found between the OLR computed on the collective partitioned networks of those students who passed (*Mdn* = 0.939) and failed (*Mdn* = 0.91); *U*(*N_a_ = 5*, *N_f_ =* 5) = 0.000, *z* = −2.611, *p* = 0.008. This result shows that the OLR can differentiate between passing students and those with poor learning performance, as was found in previous research [8]. Moreover, a significant difference in the OLR computed on individual learning pathway networks was found between students who passed (*Mdn* = 0.669) and failed (*Mdn* = 0.591); *U*(*N_a_ = 373*, *N_f_ =* 51) = 4513.5, *z* = −1, *p <* 0.001, as shown in Figure 7.

**Figure 8:**
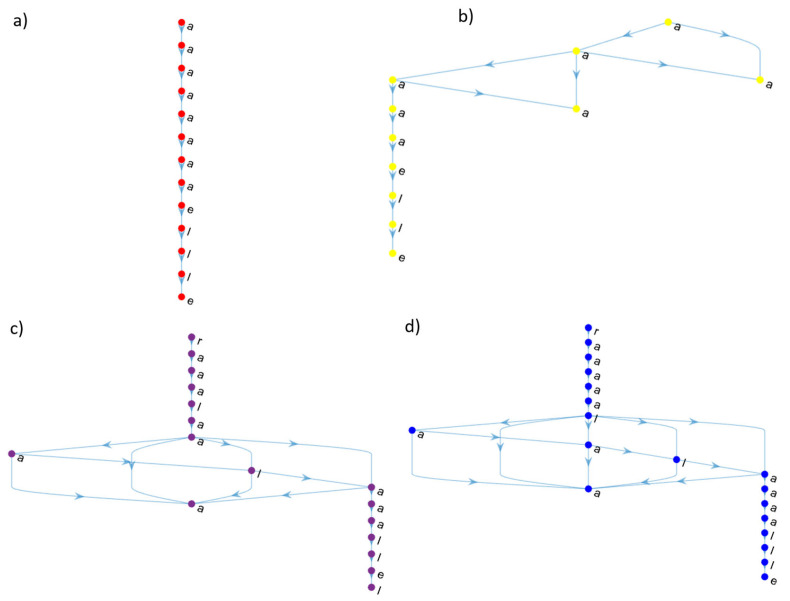
The student’s learning pathways, those of (**a**,**b**), obtained a final grade of seven and (**c**,**d**) of nine. A = activity, e = examination, l = lesson, r = reading.

A Pearson’s coefficient was calculated to assess the correlation between the OLR of individual learning pathways networks and students’ final grade *r*(424) = 0.476, *p* <0.001. Similarly, the Pearson’s coefficient was calculated between the OLR of the collective partitioned learning pathway networks and the average final grade of the students in each partition (as shown in Table 1) *r*(10) = 0.754, *p* = 0.012. The adjusted correlation coefficient (adj *R*^2^) of the linear model of the OLR of the individual learning pathways networks and students’ final grades was 0.225, *F*(1,422) = 123.903, *p <* 0.001. The regression coefficient (β = 0.476, *p <* 0.001) indicates that the final grade increases as OLR. Meanwhile, the collective partitioned learning pathway networks were adj *R*^2^ = 0.515, *F*(1,8) = 10.542, *p* = 0.012. Similarly, the regression coefficient is positive (β = 0.754, *p* = 0.012). These results suggest that a strong relationship emerges from individual learning pathways (effect size *f^2^* = 0.293) analysis to collective ones (effect size *f^2^* = 1.317) [29]. Furthermore, a complex topology emerges from the individual learning pathways when they are gathered to construct the collective ones, although the individual ones are sequential revisions with a few bifurcations, as shown in Figure 2 and Figure 8. The complex topology of collective learning pathways emerges from a non-additive process of the individual learning pathways, as the collective pathways are non-additive systems.

### 5.2. Equifinality and Learning Performance

The individual learning pathways differ from the others, but the final grade is similar or equal, as shown in Figure 8, showing equifinality.

**Figure 9:**
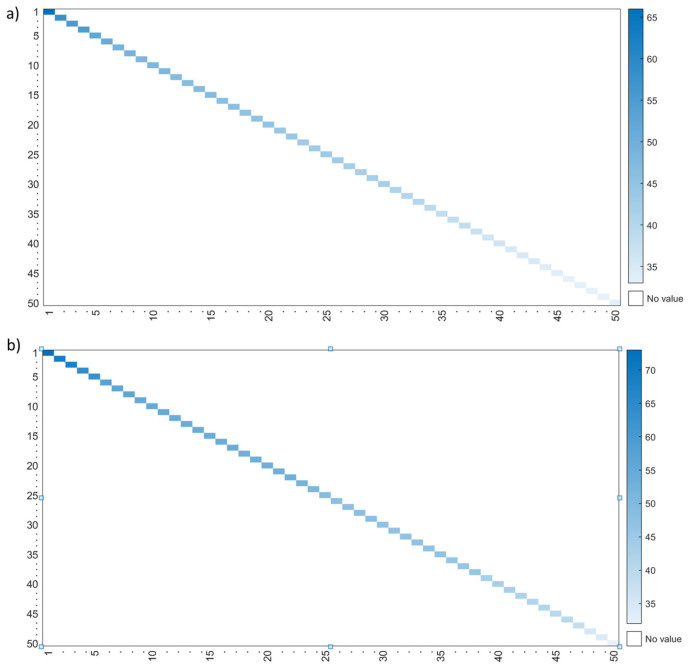
Levenshtein distance between students who (**a**) passed and (**b**) failed the course and the expected learning pathway.

Since it is not easy to compare the individual learning pathway networks, fractal node extraction was employed to obtain a decreasing ordered list of relevance, as was described in the previous section. The Levenshtein distance significantly differed between students who passed (*Mdn* = 44) and those who failed (*Mdn* = 49); *U*(*N_a_ =* 373, *N_f_ =* 51) = 4943, *z* = −5.573, *p <* 0.001. The mean distances of the learning pathways of the students who passed (44) and failed the course (49) were far from the expected learning pathway designed by the faculty members and were higher for failed students. Moreover, the minimum distance for successful students was 31 and 30 for failed students. These values suggest that not even one student reviewed the learning material in the way that it was designed. Figure 9 shows the Levenshtein distance between sequences of students who (a) passed and (b) failed the course and the expected learning pathway. Intense blue in Figure 9a,b indicates that many deletions and insertions were performed to transform the current sequence into the expected one to review the course content. Moreover, Figure 9b shows many more sequences with intense blue than Figure 9a. Hence, students who failed the course did not browse the learning material as expected.

**Figure 10:**
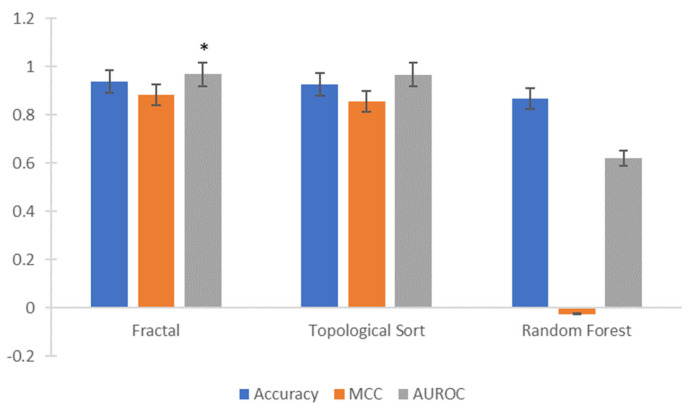
The accuracy of the Matthews Correlation Coefficient (MCC), and Area Under the Receiver Operating characteristic Curve (AUROC) for the classification of learning performance by the LSTM network. * Statistically different *p >* 0.05.

The accuracy, area under the receiver operating characteristic curve, and the Matthews correlation of LSTM, trained and tested with the list of encoded nodes extracted from the individual learning pathways, using a fractal approach, were 0.94, 0.969 and 0.884, respectively. Similarly, the LSTM was trained and tested using the nodes obtained by a topological sort of individual networks. The main difference between the extraction methods was that the fractal method obtained a list of nodes, usually less than the total nodes in the network; while, in the topological sort, the number of nodes was precisely the same as the learning pathway. The accuracy (*Mdn* = 0.927) obtained by the topological sort (*t*) was lower than that by the fractal (*f*) method (*Mdn* = 0.94); *U*(*N_t_ =* 500, *N_f_ =* 500) = 93,606, *z* = −6.957, *p <* 0.0001. The area under the receiver operating characteristic curve was higher for the fractal method (*Mdn =* 0.969) than for the topological sort (*Mdn =* 0.957); *U*(*N_t_ =* 500, *N_f_ =* 500) = 240,337.5, *z* = −2.170, *p* = 0.03. The Matthews correlation was analysed, and the Mann–Whitney U test showed that the fractal method (*Mdn =* 0.884) obtained a higher value than did the topological sort (*Mdn* = 0.857); *U*(*N_t_ =* 500, *N_f_ =* 500) = 212,427.5, *z* = −8.296, *p <* 0.0001, as shown in Figure 10. Thus, the equifinality presented in individual learning pathways is useful for classifying the students’ learning performance. Furthermore, the fractal node extraction approach outperformed the topological sort in classifying the learning performance.

Moreover, the random forest algorithm was trained and tested to compare the accuracy, area under the receiver operating characteristic curve and Matthews correlation with those obtained by classifying the individual learning pathways (extracted by the fractal method) using the LSTM network. Because the random forest cannot process a sequence of encoded nodes, each sequence was split into several attributes. For example, the sequences extracted from the learning pathways of Figure 8 are in Table 2. The random forest classification was performed using a ten-fold cross-validation, as carried out on the LSTM network.

The accuracy (*Mdn* = 0.87) obtained by the random forest algorithm^®^ was lower than the fractal method (*f*) (*Mdn* = 0.94); *U*(*N_r_ =* 500, *N_f_ =* 500) = 1209, *z* = −27.259, *p <* 0.0001. The area under the receiver operating characteristic curve was higher for the fractal method (*Mdn =* 0.969) than for the random forest (*Mdn =* 0.62); *U*(*N_r_ =* 500, *N_f_ =* 500) = 10, *z* = −27.372, *p <* 0.0001. Moreover, the Matthews correlation was analysed, and the Mann–Whitney U test showed that the fractal method (*Mdn =* 0.884) obtained a higher value than the random forest (*Mdn* = −0.025); *U*(*N_t_ =* 500, *N_f_ =* 500) = 0.0, *z* = −21.842, *p <* 0.0001, as shown in Figure 10. The low value of the Matthews correlation means that random forest incorrectly classified most positive and negative instances, and most of its positive and negative predictions were also incorrect [30,31]. These low results show that the random forest cannot learn how the sequences differentiate the students’ learning performance.

## 6. Conclusions

This research provides empirical evidence that students’ learning pathways have the properties of emergence and non-additivity in a macro-level analysis; meanwhile, equifinality is present in a micro-level inspection. A strong correlation between OLR and learning performance emerges from individual learning (micro-level) to collective (macro-level) pathways. The correlation between OLR and the final grade is positive and is statistically different in both micro-level (individual learning pathways) and macro-level (collective learning pathways) analyses for students who passed and failed. Furthermore, the complex topology of collective pathways emerges from the individual ones in a non-additive process; thus, the collective learning pathways are non-additive systems.

Equifinality means reaching the same ending by traversing different pathways but having different experiences. This property of dynamic complex systems is presented in the students’ learning pathways that contain different patterns of reviewing material but get a similar final grade. The distance between these learning pathways and the expected ways of reviewing materials can be measured by extracting the relevant nodes by the fractal method. The results show that the navigation by the students through the material differs from what was expected. The learning pathways of students who failed the course are far from the designed route. Finally, the relevant nodes extracted by the fractal method show good accuracy and area under the receiver operating characteristic curve and an acceptable Matthews correlation in classifying the learning performance of individual learning pathways. Based on the evidence presented in this article, the learning pathways have the property of emergence, equifinality and non-additivity that characterise dynamic complex systems.

The relevant pathway network (as shown in Figure 4) could be a powerful tool for future research on improving the review of learning material, since it contains the essential node. For example, the trained LSTM can generate a new pathway that contains lessons, quizzes, and learning activities extracted from the individual pathways. It could pave the way for further comparative analysis between relevant and collective pathways. These tools can offer a new view of learning pathways to researchers and scholars focused on instructional design. Moreover, an exciting future research direction is proving that learning pathways are a fractal system. A limitation of this study is the application in a single university faculty. It will be beneficial to carry out a similar study in other universities and countries and in different subject areas to validate the results presented here.

## Figures and Tables

**Table 1:** The semester and participants in the study.

Semester	Partition	Short Name	Students Enrolled	Final Grade (Mean)
January–July 2020	A	JJ2020A	33	8.73
	F	JJ2020F	9	2.33
August–December 2020	A	AD2020A	68	8.87
	F	AD2020F	7	2.29
January–July 2021	A	JJ2021A	107	8.21
	F	JJ2021F	10	4.6
August–December 2021	A	AD2021A	81	7.73
	F	AD2021F	20	3.05
January–July 2020	A	JJ2022A	84	8.13
	F	JJ2022F	5	2.4

**Table 2:** The sequences extracted from learning pathways are split into several attributes to train and test the random forest. The learning pathways are shown in Figure 8. - means no value.

Pathway	A1	A2	A3	A4	A5	A6	A7	A8	A9	A10	A11	A12	A13
Figure 8a	a	a	a	a	a	a	a	a	e	l	l	l	e
Figure 8b	a	a	l	a	a	e	e	-	-	-	-	-	-
Figure 8c	a	a	a	a	l	a	a	a	l	l	-	-	-
Figure 8d	l	a	a	a	l	a	a	a	a	l	e	-	-

## Data Availability

The data that support the findings of this study are available from the corresponding author upon reasonable request.

## Supplementary Materials

The following supporting information can be downloaded at: https://www.mdpi.com/article/10.3390/e25020291/s1, Figure S1: The box-covering implementation example of (**a**) a graph. (**b**) The result of the box number starting from node three for a box size one. (**c**) The result of the box number for a box size two. (**d**) The box assignment for the six nodes, varying the box size from one to five.
