# Supplementary material for: Learning Pathways and Students Performance: A Dynamic Complex System
Source: Entropy (Basel). 2023 Feb 3;25(2):291. doi: 10.3390/e25020291 (PMC9955738; doi:10.3390/e25020291)
Supplement: Supplementary file 1 [file entropy-25-00291-s001.zip › entropy-2116588-supplementary.pdf]

# Learning Pathways and Students Performance in Higher Education: A Complex System

## Suplementary material

### Box-covering implementation

A brief example of the computation of the box-covering code (see Code section) on the graph of Figure S1 (a) (represented by the adjacency matrix A) starts randomly selecting node three as the initial one (line 19 of the code). The column and row three are swapped with the column and row one (lines 20-42) of adjacency matrix A since the code takes the initial node as that in the first column and row. The NumberBoxes (line 43) function computes the minimum number of boxes to cover the network; the size of the boxes is between one to the diameter+1 of the network. In our example, the diameter is four. This function needs the number of nodes (nnodes), the diameter+1 of the networks and a squared matrix (that contains the shortest path distance from two nodes in the network).

a)

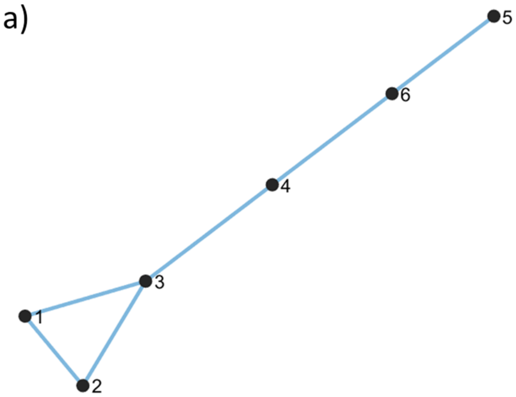


b)

| Node \ Box size | 1 | 2 | 3 | 4 | 5 |
|---|---|---|---|---|---|
| 1 | 0 | 0 | 0 | 0 | 0 |
| 2 | **1** | -1 | -1 | -1 | -1 |
| 3 | -1 | -1 | -1 | -1 | -1 |
| 4 | -1 | -1 | -1 | -1 | -1 |
| 5 | -1 | -1 | -1 | -1 | -1 |
| 6 | -1 | -1 | -1 | -1 | -1 |

c)

| Node \ Box size | 1 | 2 | 3 | 4 | 5 |
|---|---|---|---|---|---|
| 1 | 0 | 0 | 0 | 0 | 0 |
| 2 | 1 | **0** | -1 | -1 | -1 |
| 3 | -1 | -1 | -1 | -1 | -1 |
| 4 | -1 | -1 | -1 | -1 | -1 |
| 5 | -1 | -1 | -1 | -1 | -1 |
| 6 | -1 | -1 | -1 | -1 | -1 |

d)

| Node \ Box size | 1 | 2 | 3 | 4 | 5 |
|---|---|---|---|---|---|
| 1 | 0 | 0 | 0 | 0 | 0 |
| 2 | 1 | 0 | 0 | 0 | 0 |
| 3 | 2 | 0 | 0 | 0 | 0 |
| 4 | 3 | 1 | 0 | 0 | 0 |
| 5 | 4 | 2 | 1 | 1 | 0 |
| 6 | 5 | 1 | 1 | 0 | 0 |

**Figure S1.** The box-covering implementation example of a) a graph. b) The result of the box number starting from node three for a box size one. c) The result of the box number for a box size two. d) The box assignment for the six nodes, varying the box size from one to five.

This function computes the Cmin ($n$ x $m$ matrix, where $n$ is the number of nodes and $m$ =diameter+1) that summarises the box to that one node belongs given a box size. For example, column one of Cmin

contains a set of integers in each entry representing the box (of size one) to which a given node(row) belongs. It is a trivial case where each node belongs to a different box (of size one); thus, column vector one will contain *n* different numbers representing the boxes; in our example, six boxes of size one cover the network. The computation of the Cmin by the code starts initializing the matrix with -1 (lines 66-67). The start node always is assigned to the box with the number zero for every box size; thus, row one of the Cmin matrix is set to zero (line 69-71). Next, a box for the rest of the nodes is assigned (loop of line 72) for every box size (loop of line 74). It is equivalent to filling the row vector with the corresponding box starting from node two. The unusedcolor function carries out this task. Following our example, the current node is the two (line 73), and the box size is one (lb=1, line 74), so the unusedcolor function obtains the distance to the other nodes numbered less than the current node (loop of line 94 ). If the distance is equal to or greater than the size of the box (line 98), the current node (two) must be assigned to a different box (using the next integer) (lines 99 and 100). In our code, the vector usedcolors stores the index of the boxes that have been used. In our example, the distance between nodes two and three (the node tree was swapped in the adjacency matrix with node one) is one, so node two must be assigned to the next box. Since box zero was assigned to node three (this value was marked in usedcolors vector), the next box is that numbered with one (obtained by lines 109-111). Next, this value is assigned in the Cmin(2,1)=1, as shown in Figure S1 (b).

Now, the box size is increased (lb=2, line 72); thus, the distance between nodes two and three fails the condition of line 95; thus, both nodes belong to the same box; in this case, box zero; hence Cmin(2,2)=0, see Figure S1(C). The lb is increased again (lb=3, line 72) until lb=5; for these steps, the distance between node two and three is checked and fail the condition of line 95, so node 2 belongs to box zero for lb=[3,5]. The current node is moved to three (line 73 and lb=1 line 72) (numbered as 1 in Figure S1 (a) because of swapping). In this step of the loop of line 72, the unusedcolor function checks the distance between nodes 1,2,3 that are equal to one and equal to lb=1. Node three must belong to the next box available, the number two; see Figure S1 (d). The previous steps are repeated for the remaining nodes to produce the result in Figure S1 (d). The max value plus one of each column of Cmin is the minimum number of boxes of size equal to the number of the column (lines 50-51)

## Code

```
1 %Input%
2 % A: adjacency matrix of an undirected network
3 %Output %
4 %l: the diameter of the boxes [1, Diameter+1]
5 %Nb: the minimum number of boxes of diameter l to cover the network
6 %Cmin matrix that indicate the box of each node
```

```
function [l,Nb,Cmin]=boxcovering(A)
 nnodes=size(A,1);
 G=graph(A);
 d=distances(G,'Method','unweighted');
 diameter=max(d(isfinite(d))); %  bug max(d(:));
 Aor=A;
 dor=d;
 A=Aor;
 d=dor;

 %we simulate that  the first node was picked out randomly
 % by changing the adjacency matrix row and column
 rvertex=randi(nnodes);
 d1=d(1);
 d2=d(rvertex);
 d(1)=d2;
 d(rvertex)=d1;
 for i=1:nnodes
     r1=A(1,i);
     rr=A(rvertex,i);
     A(1,i)=rr;
     A(rvertex,i)=r1;
 end
 for i=1:nnodes
     c1=A(i,1);
     cr=A(i,rvertex);
     A(i,1)=cr;
     A(i,rvertex)=c1;
 end

 firstc=d(:,1);
 d(:,1)=d(:,rvertex);
 d(:,rvertex)=firstc;
 firstr=d(1,:);
 d(1,:)=d(rvertex,:);
 d(rvertex,:)=firstr;
 Cmin=NumberBoxes(nnodes,diameter+1,d);
 %change the vertex to the final position
 for w=1:length(Cmin(1,:))
     aux=Cmin(1,w);
     Cmin(1,w)=Cmin(rvertex,w);
     Cmin(rvertex,w)=aux;
 end
 Nb=max(Cmin);
 Nb=Nb+1;
 l=1:length(Nb);
 l=transpose(l);
 Nb=transpose(Nb);
end
%computes the number of boxes from l=1 to lbmax
%Input%
%% nnodes : number of nodes in the network
%%lbmax : max diameter of the boxes, usually diameter +1
%d: minimum distance between nodes nodes
%Output%
```

```
%colors:Matrix of size= number of nodes X diamter+1.  Indicates the nodes (rows)
% colour (box) for a given distance l (column)
function [colors]= NumberBoxes(nnodes,lbmax,d)
%creates matrix #nodes*lbmax distance lbmax
Cmin=zeros(nnodes,lbmax); %note that -1 indicates no color
Cmin=Cmin-1;%this is to remark that value -1 means that a node have not been colored yet
%coloring actual vertex with 0
for i=1: lbmax
    Cmin(1,i)=0;
end
for i=2:nnodes
    actualvertex=i;
    for lb=1:lbmax
        ucolor=unusedcolor(actualvertex,lb,d,Cmin);
        if ucolor~=-1 %uclor =-1  means that any distance lij was less than lb
            Cmin(actualvertex,lb)=ucolor;
        else
            Cmin(actualvertex,lb)=0;
        end
    end
end
colors=Cmin;
end

%finds the unused color for a given lb
 function  color= unusedcolor( i, lb, distances,Cmin)
        nnodes=size(Cmin);
        nnodes=nnodes(1,1);
        color=-1;
        distancechecked=0;
        usedcolors=zeros(nnodes,1);
        ucolor=-1;
        for j=1:i-1
            if isinf(distances(i,j))
                continue;
            end
            if distances(i,j)>=lb
                   ucolor=Cmin(j,lb);
                   usedcolors(ucolor+1,1)=1;
                   distancechecked=distancechecked+1;
            end
        end
        %search for the first value of usedcolor set to 0
       if distancechecked==0 %any distance was >=lb so color must be 0
           color=0;
       else
           for i=1:nnodes
               if usedcolors(i,1)~=1 %we picked out the first color to be used
                   color=i-1;
                   break;
               end
           end
       end
 end
```

```
%input
%Table LogData: data of a semestral course with the following variables
%id unique identifier of a row
%userid: unique identification for a student
%idelement:  identification for learning material in LMS
%name: name of learning material; idelement, name is a primary key
%star: start time for reviewing, solving, open the learning material
%endt: end time, the opposite of the start variable
%%%
%output
%LP: Learning pathway for each student by an adjacency matrix of directed
%CLP: Collective Learning Pathway
%network, they contain all the learning activities in the course although
%they have not been reviewed by a student
%Name: The name of the nodes of each network.
%Id The id of the row from log data

function [LP,CLP, Name, Id]=buildlearningpath(LogData)
  Id=unique(LogData.userid(:));
  Name=LogData(:,["idelement","name"]);
  Name=unique(Name,"rows");
  nodes=height(Name);
  for i=1:length(Id)
    A=sparse(nodes,nodes);
    studentLD=LogData(LogData.userid(:)==Id(i),:);
    [~,ia]=unique(studentLD.idelement);
    namei=studentLD(ia,[3,4]); %names node of individual network
    studentLD=sortrows(studentLD,["start","endt"]); %order rows by start=5 and end=6
    if height(studentLD)>=1
      item=studentLD(1,:);
      [A,items]=sequential(item,studentLD,Name,A);
      for j=1:height(studentLD)
        [A,items]=parallel(studentLD(j,:),studentLD(j:end,:),Name,A);
      end
    end
    LP{i}=A;
  end
  for i=1:length(LP)
    if i==1 CLP=LP{i};
    else CLP=CLP+LP{i};
    end
  end
end

%search for parallel items based on start and end date
function [A,items]=parallel(item,LogData,Name,A)
items=LogData(LogData.start(:)>=item.start(1) &...
  LogData.start(:)<item.endt(1) & LogData.id(:)~=item.id(1), :);
  %while(~isempty(LogData))
  [ixfrom,~]=find(Name.idelement(:)==item.idelement(1)...
    & Name.name(:)==item.name(1));
  if height(items)>=1
    itemsc1=items(items.endt(:)>=item.endt(1),:);
    itemsc2=items(items.endt(:)<item.endt(1),:);
```

```
        itemsc3=items(items.start(:)>item.start(1) & ...
            items.endt(:)==item.endt(1),:);
        itemsc4=items(items.start(:)==item.start(1) & ...
            items.endt(:)==item.endt(1),:);
        for i=1:height(itemsc1)%case 1
            [ixto,~]=find(Name.idelement(:)==itemsc1.idelement(i)...
                & Name.name(:)==itemsc1.name(1));
            A(ixfrom,ixto)=A(ixfrom,ixto)+1;
            itemc1=itemsc1(i,:);
            nextix=nextnode(itemc1,LogData,Name);
            if nextix~=-1
                A(ixto,nextix)=A(ixfrom,ixto)+1;
                [A,items]=parallel(itemc1,LogData,Name,A);
                [A,items]=sequential(itemc1,LogData,Name,A);
            end
        end
        for i=1:height(itemsc2) %case 2
            [ixto,~]=find(Name.idelement(:)==itemsc2.idelement(i)...
              & Name.name(:)==itemsc2.name(i));
            A(ixfrom,ixto)=A(ixfrom,ixto)+1;
            A(ixto,ixfrom)=A(ixto,ixfrom)+1;
            [A,items]=parallel(itemsc2(i,:),LogData,Name,A);
            [A,items]=sequential(itemsc2(i,:),LogData,Name,A);
        end
        for i=1:height(itemsc3) %case 3
            [ixto,~]=find(Name.idelement(:)==itemsc3.idelement(i)...
                && Name.name(:)==itemsc3.name(1));
            A(ixfrom,ixto)=A(ixfrom,ixto)+1;
            A(ixto,ixfrom)=A(ixto,ixfrom)+1;
            [A,items]=parallel(itemsc3(i,:),LogData,Name,A);
            [A,items]=sequential(itemsc3(i,:),LogData,Name,A);
        end
        for i=1:height(itemsc4)%case 4
            [ixto,~]=find(Name.idelement(:)==itemsc4.idelement(i)...
                & Name.name(:)==itemsc4.name(1));
            A(ixfrom,ixto)=A(ixfrom,ixto)+1;
            A(ixto,ixfrom)=A(ixto,ixfrom)+1;
            [A,items]=parallel(itemsc4(i,:),LogData,Name,A);
            [A,items]=sequential(itemsc4(i,:),LogData,Name,A);
        end
    end
    %end
end
%search for non overlapping items based on start and end date
function [A,items]=sequential(item,LogData,Name,A)
    items=LogData(LogData.start(:)>item.start(1) & ...
        LogData.start(:)>=item.endt(1)& LogData.id(:)~=item.id(1),:);
    while(~isempty(items))
        [ixfrom,~]=find(Name.idelement(:)==item.idelement(1)...
            & Name.name(:)==item.name(1));
        if height(items)>=1
            [ixto,~]=find(Name.idelement(:)==items.idelement(1)...
                & Name.name(:)==items.name(1));
            A(ixfrom,ixto)=A(ixfrom,ixto)+1;
            [A,items]=sequential(items(1,:),items,Name,A); %before LogData instead items
        end
```

```
    end
end

function nextix=nextnode(item,LogData,Name)
nexnode=LogData(LogData.start(:)>item.start(1) &...
    LogData.start(:)>=item.endt(1)& LogData.id(:)~=item.id(1),:);
if(~isempty(nexnode))
    [nextix,~]=find(Name.idelement(:)==nexnode.idelement(1)...
        & Name.name(:)==nexnode.name(1));
else
    nextix=-1; %not found
end
end
```
